# The Design, Development, and Testing of a Virtual Reality Device for Upper Limb Training in People With Multiple Sclerosis: Single-Center Feasibility Study

**DOI:** 10.2196/36288

**Published:** 2022-09-12

**Authors:** Alon Kalron, Lior Frid, Iliya Fonkatz, Shay Menascu, Mark Dolev, David Magalashvili, Anat Achiron

**Affiliations:** 1 Department of Physical Therapy School of Health Professions, Sackler Faculty of Medicine Tel-Aviv University Tel-Aviv Israel; 2 Multiple Sclerosis Center Sheba Medical Center Tel Hashomer Israel; 3 Sackler Faculty of Medicine Tel-Aviv University Tel-Aviv Israel

**Keywords:** virtual reality, rehabilitation, feasibility, upper limb, multiple sclerosis

## Abstract

**Background:**

Multiple sclerosis (MS) is a common nontraumatic, neurological, disabling disease that often presents with upper limb dysfunction. Exercise training has resulted in improvement for patients; however, there can be a lack of compliance due to access because of location and lack of MS experts. Virtual reality (VR) is a promising technology that can offer exercise therapy/rehabilitation at a distance. This type of remote training can be motivational and effective for patients with MS and can improve range of motion and muscle strength for those with upper limb dysfunction.

**Objective:**

The aim of this study is to evaluate the safety and feasibility of the XRHealth software and the Oculus Rift Station for patients with MS with upper limb motor dysfunction.

**Methods:**

A single-center, prospective, feasibility study was conducted with patients with MS who had upper limb motor dysfunction. Patients participated in a single 45-minute digital environment session with VR and completed a questionnaire about the quality of the training and fatigability. The clinician also completed a questionnaire to evaluate the suitability and safety of the training.

**Results:**

Overall, 30 patients were enrolled between the ages of 20 and 81 years. Patients reported that the training sessions within the digital environment were helpful, challenging, fun, and simple to understand, and that they would be willing to repeat the sessions again. The physical therapist that oversaw the patients reported that the training was suitable for 87% (n=26) of the patients. Anticipated adverse events were fatigue, temporary dizziness, and temporary nausea. The operator complications included that the cable of the head-mounted display interrupted the training (n=2, 7%) and fatigue that caused cessation of the VR training session (n=2, 7%). No serious adverse events were reported.

**Conclusions:**

These preliminary results demonstrated that the use of the XRHealth software and Oculus Rift Station platform is feasible, safe, and engaging for patients, and has the potential to improve the functionality of the upper limbs in patients with MS. This study provides support for future studies of implementing a series of training sessions with virtual reality in a home-based environment.

## Introduction

### Background

Multiple sclerosis (MS) is a chronic progressing demyelinating disease of the central nervous system [1]. The age at which MS is generally diagnosed is between the ages of 20-40 years, with a higher prevalence in female patients than male patients [2]. MS is the most common nontraumatic neurological disabling disease affecting young adults with an incidence of approximately 1 in 1000 [2], which affects approximately 2.8 million people worldwide (35.9 per 100,000 population) [3].

In patients with MS, upper limb dysfunction is common and occurs in approximately 50% to 76% of patients [4,5]. The presentation of upper limb dysfunction can be variable in patients with a range of physical symptoms presenting as tremor, lack of movement coordination, or muscle weakness [1,6,7]. These symptoms can lead to a decreased quality of life (QoL) due to poor health status, psychological difficulties, depressed mood and cognitive difficulties [8], increased mortality, restriction of activities [9,10], and dependency on others [11]. Some of the most frequent challenges that patients with MS face is dexterity, activities of daily living, and limitations in the upper limbs [12]. Treatment approaches for MS typically involve pharmacological intervention and rehabilitation treatments [13-15].

Symptoms of MS can range from visual impairment; dysarthria and dysphagia; impairment of coordination and balance; ataxia; sensory impairment; and bladder, bowel and sexual dysfunction [1,16-19]. Exercise training for MS and those with upper limb dysfunction has demonstrated significant benefit [20-24] and has been able to mitigate symptoms and decrease dependency on others [15,22,23].

The major reasons for lack of compliance in patients with MS are the inability to access exercise programs because patients live in a rural area or location that lack MS experts that support traditional upper limb exercise programs. Patient transportation and access can be limited due to time commitments, vehicle access, and simply not having the health and physical ability to travel to a hospital for physical rehabilitation. Another major obstacle is the cost of care that is prohibitive for patients. The COVID-19 pandemic has highlighted the need to provide therapy/rehabilitation from a distance, mainly due to travel restrictions, access, and risk of infection [25]. Since rehabilitation can be especially difficult for the patient due to time and physical demands, loss of motivation and compliance often occur; therefore, it is critical to develop innovative new methods to keep patients motivated and improve QoL.

A method to circumvent these challenges and support exercise training in the home of patients and increase physical activity and functionality, thereby improving QoL, is telerehabilitation [26]. Therapy is delivered remotely with telerehabilitation outside traditional hospital settings, providing access to patients who have problems with mobility and travel restrictions. A promising new component is virtual reality (VR) for patients. VR provides a 3D-simulated environment with a computer interacting with a number of electronic accessories that can motivate patients to do repetitive tasks with sensory and visual feedback [27]. The level of immersion with VR may vary, ranging from nonimmersive (eg, video games) to fully immersive, where the user must have proper VR glasses or a head-mounted display such as the HTC Vive or Oculus Rift. There are different ideas on the classification of VR to date, and a classification remains to be standardized between studies [28]. Immersive VR has been applied to psychological therapy for many disorders including posttraumatic stress disorder, borderline personality disorder, schizophrenia, and other psychological disorders [29]. Patients can practice functional tasks such as shopping or crossing the street in a virtual environment [27]. The benefits of VR have been established in multiple applications including pain management and reduction [30-34], mental health support [33-36], and rehabilitation [32]. In MS, the application of VR has improved balance, posture, motor control, coordination, and gait [37-40] as well as improved motor function in the upper limbs [41]. A recent study demonstrated an improvement in manual dexterity for patients with MS using game-based VR video capture training [42]. The controlled environment of immersive VR can lead to improved patient outcomes compared with conventional therapy in different types of motor impairments including hemiparesis caused by Parkinson disease, MS, cerebral palsy, and stroke [28,29,41,43]. Despite the promising results, VR is not widely implemented, resulting in an unmet medical need for a growing patient population.

Currently, XRHealth software is cleared for marketing in the United States by the Food and Drug Administration, Europe (EC certification), and Israel (AMAR). The software along with the Oculus Rift Station guides patients through exercises according to an individualized treatment plan from their medical practitioner. The Oculus Rift station has touch controllers and provides *df* for the movement inside a virtual environment, which includes high-end tracking of both the head and arm movements with 0.01 degrees in 90 Hz accuracy. Through the combination of the XRHealth software, the Oculus Rift Station, and a clinical therapist, the motion and movement kinetics of a patient can be tracked and provided to a clinician.

### Objectives

The objective of this pilot study is to evaluate the combination of the XRHealth software with the Oculus Rift Station for safety and feasibility in patients with MS with upper limb motor dysfunction. The quality of the resulting data from this platform was assessed for optimization and serves as preliminary data for the development and design of future studies for VR training in people with MS.

## Methods

### Ethics Approval

This is a single-center safety and feasibility study based on a single 45-minute training session. All patients provided informed consent, and institutional review board approval was obtained at the Sheba Medical Center (SMC-5207-18). This study was conducted in the Sheba Multiple Sclerosis Center, Tel-Hashomer, Israel, between 2019 and 2020.

### Patients

Patients were enrolled if they were 18 years or older, had moderate weakness in upper extremity muscles as defined by the British Medical Research Council including those with grade 4 in 2 muscle groups, or British Medical Research Council grade 3 in 1 muscle group. Patients were excluded if they had a hearing impairment, any orthopedic impairment restricting upper limb movements, or any cardiovascular disease preventing aerobic exercise.

### VR Training Platform and Device

The intervention involved 2 activities performed once within the VR environment that would require both arm and shoulder movements either while seated (Figure S1A in Multimedia Appendix 1) or standing (Figure S1B in Multimedia Appendix 1) on the setup shown in Figure S1C in Multimedia Appendix 1. This was performed with the Oculus Rift and XRHealth software. The Oculus Rift headset is similar to goggles and, when worn, allows the patient to look in any direction naturally as if in the real world. In the Oculus Rift headset, there is a tracker that constantly analyzes head movement, allowing a clinician to make adjustments for the patient in real time [44]. The XRHealth software platform has three main components: a central console, a data portal, and a control panel. In the central console, a range of different motor, cognitive, and mental experiences in the form of games and exercises can be selected and serves as a “home environment” for all XRHealth applications. The data portal is a Health Insurance Portability and Accountability Act–compliant web-based platform that clinicians can use to track and analyze data from patients, and offers longitudinal data about patient performance and progress. The VR experience can be viewed and adjusted remotely and in real time by the clinician, and is supported by the control panel.

All assessments were overseen by a physical therapist with 2 years of experience in neurohabilitation. The first activity was an active shoulder range of motion (ROM) application called “Balloon Blast.” In this VR environment, the patient uses both hands and is prompted to pop balloons with a swipe of a sword (Figure S2A in Multimedia Appendix 1). The area of shoulder activity is determined with an active ROM measurement (Figure S2B and C in Multimedia Appendix 1). After a 5-minute rest period, the patient performed the second activity, which is a motor cognitive training environment called “Color Match.” Patients wear different colored virtual gloves on both hands and are then prompted to hit light bulbs with the hand that matches the bulb color (Figure S2D in Multimedia Appendix 1). The initial settings of the “Color Match” VR environment were set to a medium level for all patients and adjusted according to the patient’s capabilities. Medium level was defined as 3.6 seconds between the moment the light turns on and then turns off for the patient to act.

### Outcome Measures for Safety and Feasibility

Each patient answered 4 questionnaires for the assessment (Supplemental 1 in Multimedia Appendix 1), and the physical therapist who oversaw the session also completed a questionnaire (Supplemental 2 in Multimedia Appendix 1). The lowest score was 1 and the highest was 4. Patient answers were averaged together for a final result. Patient questionnaires aimed to evaluate patient experience in relation to the VR platform for optimization and to inform the design of future studies. Questions included patient assessment on the level of difficulty, physical effects such as fatigue, engagement, and suitability of the VR training session.

## Results

### Patient Characteristics

A total of 30 patients with MS with upper limb motor dysfunction were enrolled and completed the training. Of those, 50% (n=15) were female patients, the mean age was 50.8 (range 20.3-81.2) years, and the mean Expanded Disability Status Scale score was 5.4 (Table 1).

**Table 1:** Patient demographic and clinical characteristics.

Characteristic		Patients (N=30), n (%)	Value		
			Mean (SD)	Range	
Age (years)		N/A^a^	50.74 (13.6)	20.3-81.2	
Female, n (%)		15 (50)	N/A	N/A	
**Multiple sclerosis type, n (%)**			N/A		N/A
	Primary progressive multiple sclerosis	3 (10)			
	Clinically isolated syndrome	3 (10)			
	Relapsing and remitting multiple sclerosis	14 (47)			
	Secondary progressive multiple sclerosis	10 (33)			
Disease duration (years)		N/A	18.6 (12.8)	0.6-58	
Expanded Disability Status Scale		N/A	5.4 (1.7)	1.5-8.0	

^a^N/A: not applicable.

### Patient Satisfaction

**Figure 1:**
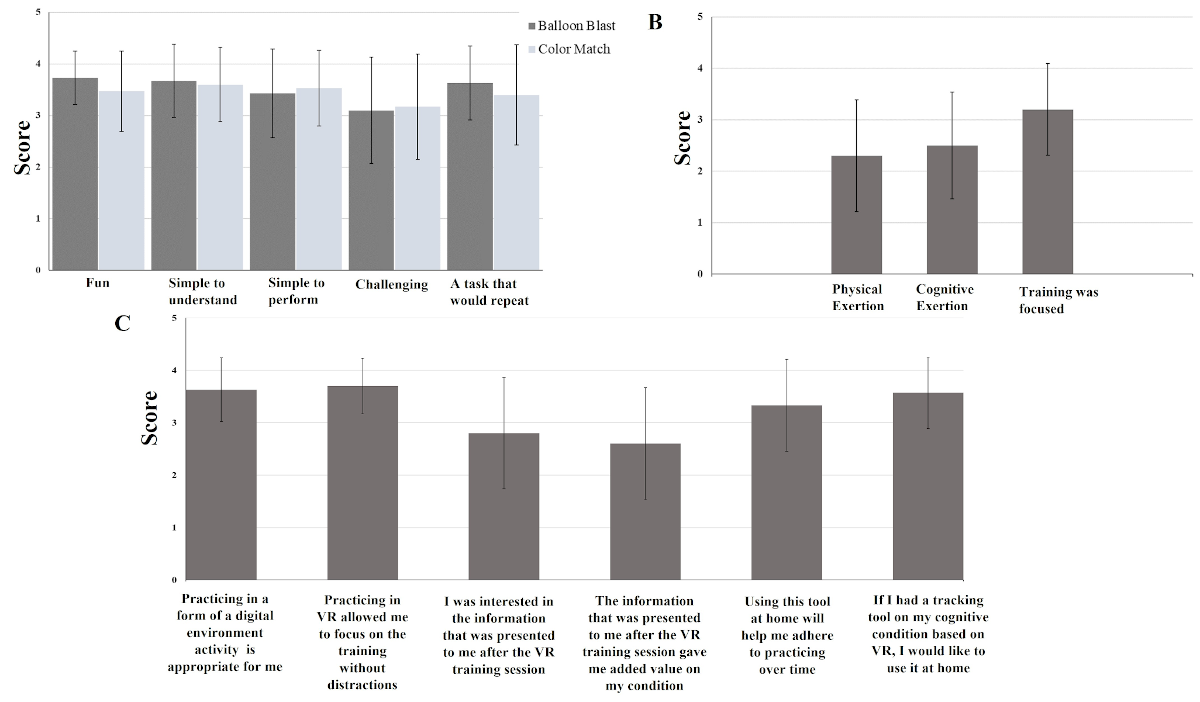
Questionnaire results: (A) self-reported satisfaction questionnaire average, (B) the overall VR experience reported by patients, and (C) statements about the session and levels of patient agreeance. VR: virtual reality.

Patients reported in the questionnaires that the “Balloon Blast” activity was fun (mean 3.73, SD 0.52), simple to understand (mean 3.67, SD 0.71), simple to perform (mean 3.43, SD 0.86), challenging (mean 3.10, SD 1.03), and a task that they would repeat (mean 3.63, SD 0.72; Figure 1A). In the “Color Match” activity, patients reported that the activity was fun (mean 3.47, SD 0.78), simple to understand (mean 3.60, SD 0.72), simple to perform (mean 3.53, SD 0.73), challenging (mean 3.17, SD 1.02), and a task that they would repeat (mean 3.40, SD 0.97; Figure 1A).

For overall VR experience satisfaction, patients reported that during the VR training session they felt physical exertion (mean 2.3, SD 1.09) and cognitive exertion (mean 2.5, SD 1.04), and that the training was focused (mean 3.2, SD 0.89; Figure 1B). Using the same scoring system, patients reported high levels of agreement with the following statements: practicing in a form of a digital environment activity is appropriate for me (mean 3.63, SD 0.61); practicing in VR allowed me to focus on the training without distractions (mean 3.70, SD 0.53); I was interested in the information that was presented to me after the VR training session (mean 2.8, SD 1.06); the information that was presented to me after the VR training session gave me added value on my condition (mean 2.6, SD 1.07); using this tool at home will help me adhere to practicing over time (mean 3.33, SD 0.88); and if I had a tracking tool on my cognitive condition based on VR, I would like to use it at home (mean 3.57, SD 0.68; Figure 1C). After completing the VR training sessions with the patients, the physical therapist reported that this was a suitable solution for people with MS (n=26, 87%).

### Safety

Reported intraoperative complications included that the cable of the head-mounted display interrupted the training (n=2, 7%) and fatigue that caused cessation of the VR training session (n=2, 7%). There were no other intraoperative complications reported. Anticipated adverse events (AEs) according to a 4-point Likert scale were fatigue (mean 1.9, SD 0.96), temporary dizziness (mean 1.57, SD 0.77), and temporary nausea (mean 1.17, SD 0.46). Of the patients that reported fatigue, 6 patients experienced light fatigue, 9 patients reported moderate fatigue, and 1 patient reported severe fatigue resulting in a score of 1. Of those that reported dizziness, 7 patients reported light dizziness and 4 patients reported moderate dizziness. In patients that reported nausea, 3 reported light nausea and 1 reported moderate nausea. No other unanticipated AEs were reported. There were no serious AEs. All the AEs reported were resolved within a short period of time.

## Discussion

### Principal Results

Overall, patients found the VR environment training session helpful and would be willing to do more. The physical therapist determined that the session was suitable for people with MS. Physical exercise therapy for patients with MS is critical in maintaining movement, mental health, and the ability to be self-sufficient [21]. This study supports moving the platform into a home-based environment, which reduces the financial and travel burden on patients while also improving health and QoL [45-48]. Furthermore, the VR digital environment is a simple patient interface in which patients reported the programs to be engaging, challenging, simple, and fun. Patients had minimal risk, and the activities were safe and tolerable. There were no serious AEs, and those that were reported were temporary.

The XRHealth platform is performed under medical supervision during training and allows the physical therapist to modify several parameters during a session that meets the specific needs of the patient. The types of assessments that can be performed include ROM, action time, response time, and omission and commission mistakes collected during a single 45-minute training session, which is then output as a graph by the XR software (Figure S2E in Multimedia Appendix 1). The ability to involve a clinician and obtain a number of data points to determine outcomes while patients are receiving care in a digital environment are substantial benefits.

### Limitations

This study is not without limitations. First, the study did not include standard measures of upper limb function or manual dexterity. In addition, future adaptations could use the standardized System Usability Scale rather than the customized questionnaire used in this study based on similar publications in the field. Furthermore, this was a small study with a single VR training session. Future larger studies are needed to investigate whether scores derived from the VR system correlate with clinical upper limb measures in people with MS and other populations with central neurological damage such as stroke or Parkinson disease. A larger and longer study with more sessions would provide the efficacy data needed to support the use of a VR environment for physical therapy in people with MS.

### Comparison With Prior Work

The use of VR technology has revealed improvements in the clinic for patients with upper limb dysfunction and stroke [49,50], and has also demonstrated benefit and favorable results in improving gait impairments and balance in people with MS [51]. Early research in 2006 examining the efficacy of VR showed improvements in gait control for patients with MS [52]. A small study of 5 patients with MS showed that VR plus passive robotic support yielded improved upper limb function and was well tolerated [53]. In 2015, another study of 30 people with MS demonstrated that VR had significant improvement in overall stability after 24 sessions [38]. There are few prior studies, and it is unclear if the use of VR training in people with MS can improve the QoL status, although these studies show a positive trend in assisting people with MS. Currently, the use of VR is limited in the clinic for patients with MS despite preliminary successes. The XRHealth platform therefore has the ability to bring this technology to a broader patient population.

At this time, there are small ongoing randomized controlled trials (RCTs) integrating VR into MS therapy for upper limb dysfunction. These include the Telerehabilitation and Multiple Sclerosis (TEAMS) pilot RCT (ClinicalTrials.gov NCT04032431) comparing conventional therapy to a home-based telerehabilitation VR program [54], a single-blinded RCT using serious games for upper limb rehabilitation (ClinicalTrials.gov NCT04171908) [42], and a randomized interventional study adding game-based VR exercises to conventional physiotherapy (ClinicalTrials.gov NCT04212689) [55]. This pilot study contributes to these studies in support of integrating VR for rehabilitation of people with MS with upper limb dysfunction.

### Conclusions

In conclusion, the XRHealth medical solution of a VR digital environment and Oculus Rift platform is a feasible and safe training system for upper limb training in people with MS. These findings pave the way for future RCTs to examine the benefits of the XRHealth VR training compared with standard care to improve functionality of the upper limbs in people with MS. Additionally, it would be noteworthy to compare VR training at a clinical facility compared to VR training telehealth, both monitored by a physical therapist. Finally, future research should examine the psychometric values of the outcome measures produced by the XRHealth platform in patients with upper limb dysfunctions.

## Appendix

Multimedia Appendix 1 Supplementary material.

## Abbreviations

AE: adverse event
MS: multiple sclerosis
QoL: quality of life
RCT: randomized controlled trial
ROM: range of motion
TEAMS: Telerehabilitation and Multiple Sclerosis
VR: virtual reality
