# Supplementary material for: The Design, Development, and Testing of a Virtual Reality Device for Upper Limb Training in People With Multiple Sclerosis: Single-Center Feasibility Study
Source: JMIR Serious Games. 2022 Sep 12;10(3):e36288. doi: 10.2196/36288 (PMC9513692; doi:10.2196/36288)

**Virtual reality training to improve upper limb motor function in multiple sclerosis: A feasibility study**

Alon Kalron^a,b^, Lior Frid^a^, Iliya Fonkatz^a^, Shay Menascu^a^, Mark Dolev^a^, David Magalashvili^a^, Anat Achiron^a,d^

^a^Multiple Sclerosis Center, Sheba Medical Center, Tel Hashomer, Israel

^b^Department of Physical Therapy, School of Health Professions, Sackler Faculty of Medicine, and Sagol School of Neurocience, Tel-Aviv University, Tel-Aviv, Israel

^c^XRHealth Ltd

^d^Sackler Faculty of Medicine, and Sagol School of Neurocience, Tel-Aviv University, Tel-Aviv, Israel

**Supplementary Material**

**Figure S1. XRHealth set up and use**

**Figure S2.** **XRHealth scenes for training**

**Supplement 1. Patient questionnaire**

**Supplement 2. Trainer-reported questionnaire**


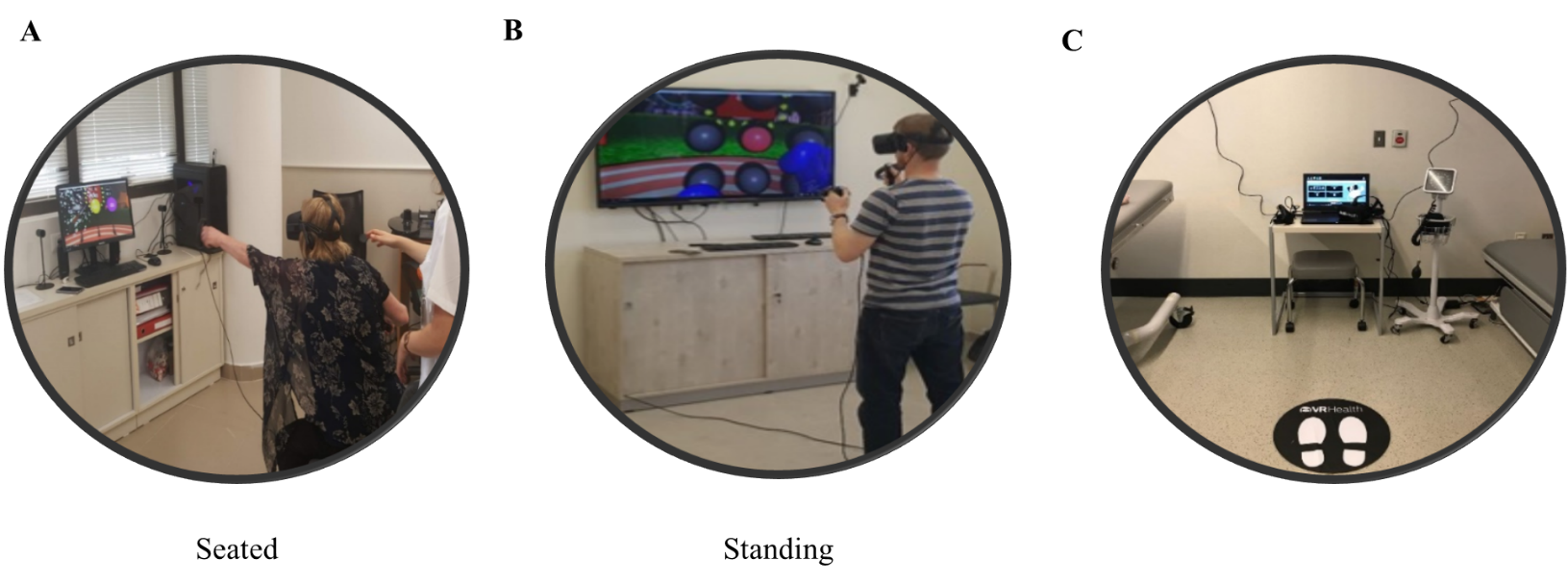


**Figure S1.** **XRHealth set-up and use**. **A**. A seated user wears the head mounted display and holds 2 hand-held sensors, while a clinician supervises throughout the training. **B**. Oculus-Rift platform and station**. C**. Positioning of the system’s components: Computer, IR sensors, headset, and controllers.

**
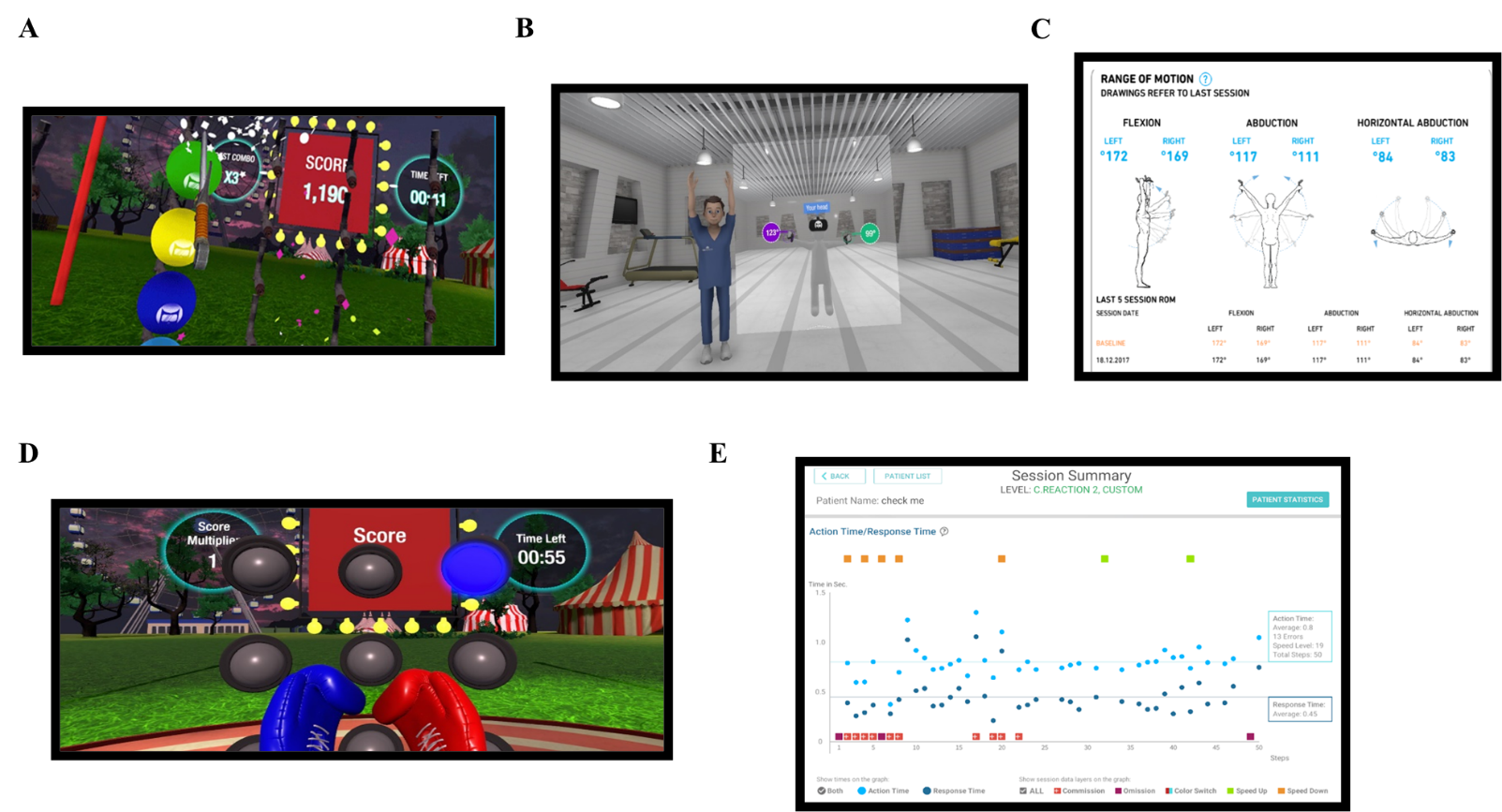
**

**Figure S2. XRHealth scenes for training. A**. Scene from VRHealth’s application “Balloon Blast”. **B.** VR Range of motion (ROM) test as performed by a VR user. **C.** ROM test results. **D**. Scene from VRHealth’s application “Color Match”. **E.** “Color Match” application session results as presented on the software interface.

**Supplement 1. Patient Questionnaire**


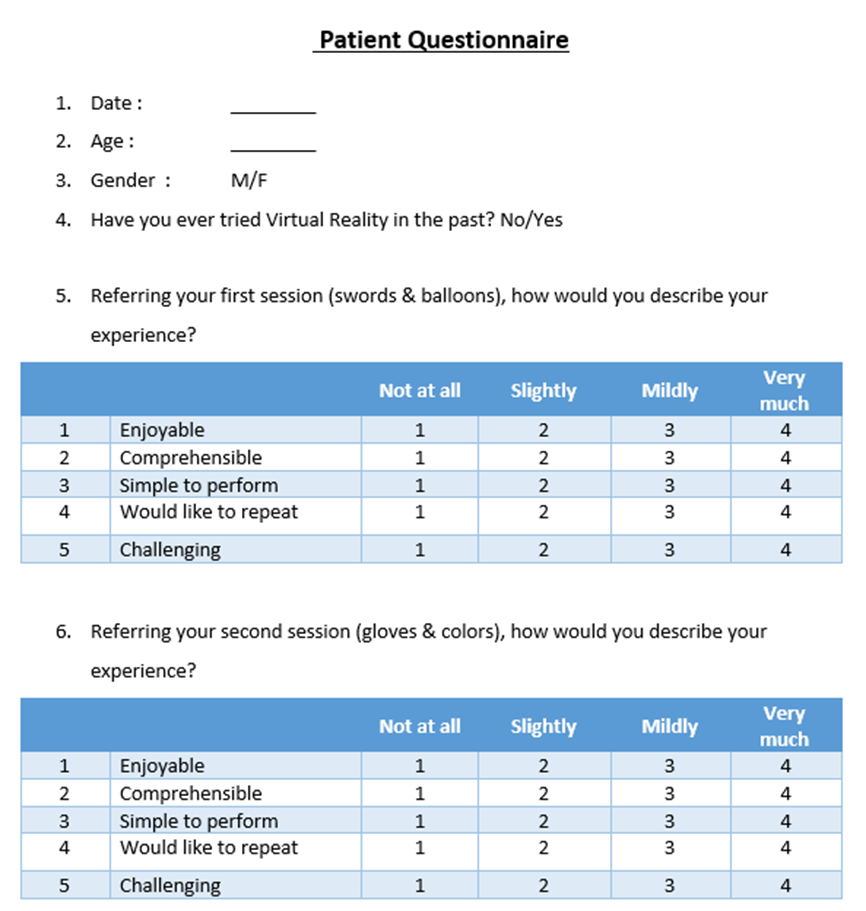


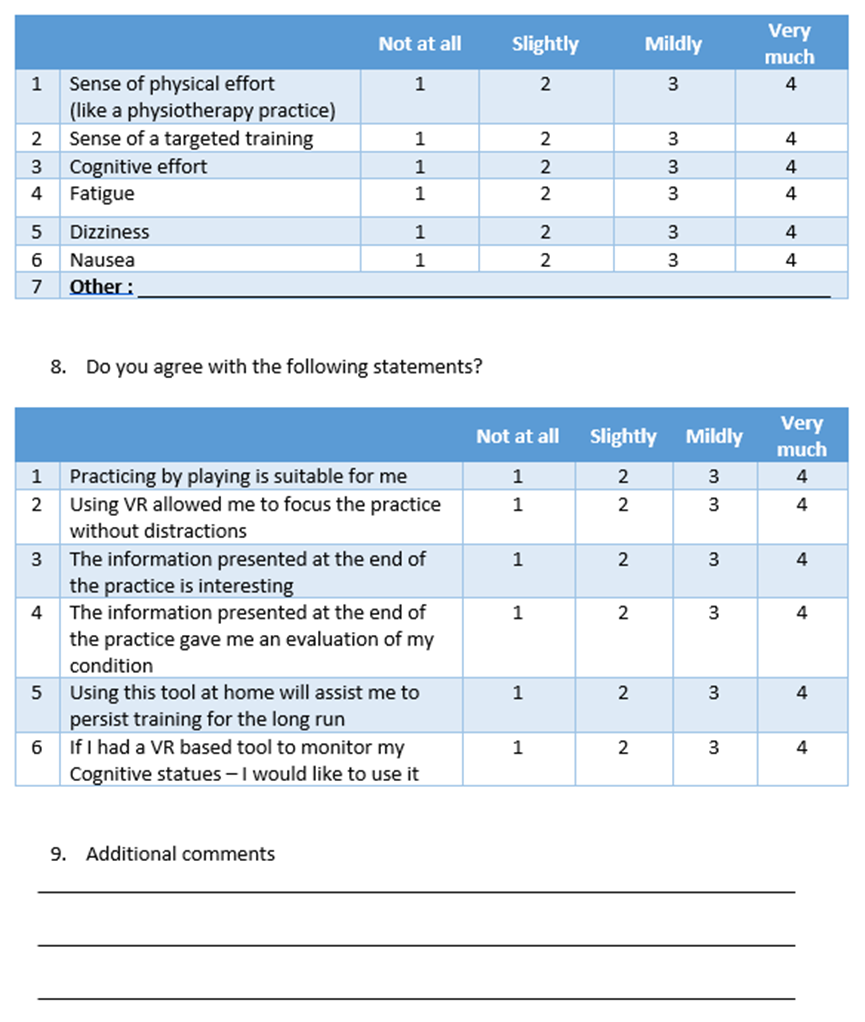


**Supplement 2. Trainer-reported questionnaire**


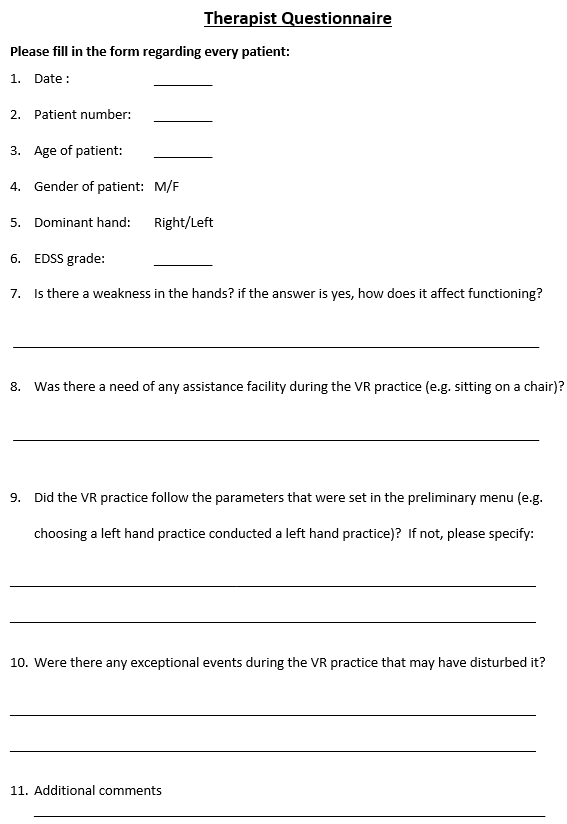

Supplement: Multimedia Appendix 1 [file games_v10i3e36288_app1.docx]
